# A comparison of citrulline and arginine for increasing exercise-induced vasodilation and blood flow

**DOI:** 10.1186/1550-2783-12-S1-P6

**Published:** 2015-09-21

**Authors:** Jordan R Moon, Roxanne M Vogel, Paul H Falcone, Matt M Mosman, Aaron C Tribby, Chad M Hughes, Jonathan D Griffin, Schyler B Tabor, Dylan J LeFever, Stephen B McChaughey, Michael P Kim, Jordan M Joy

**Affiliations:** 1 MusclePharm Sports Science Institute, Denver, CO, USA; 2 Department of Sports Exercise Science, United States Sports Academy, Daphne, AL, USA; 3 Department of Human Performance, Concordia University Chicago, River Forest, IL, USA; 4 Department of Movement Science, Grand Valley State University, Allendale, MI, USA; 5 Department of Biomedical Engineering, Widener University, Chester, PA, USA; 6 The Hospitality College, Johnson and Wales University, Denver, CO, USA; 7 Department of Human Performance and Sport, Metropolitan State University, Denver, CO, USA

## Background

One goal of supplementation has been to increase blood flow to skeletal muscle during exercise. Raw L-citrulline (RC) and raw L-arginine (RA) has often been used for its vasodilatory effects, and recently, citrulline and arginine have been bound to a whey peptide (CP and AP, respectively) to increase bioavailability. The purpose of the present study was to compare the acute hemodynamic effects of RC, CP, RA, and AP following resistance exercise in healthy, men when administered at a common, commercial dose.

## Methods

In a double-blind, crossover, placebo-controlled design, 11 recreationally-active males (28.2 ± 5.0y, 182.4 ± 5.7cm, 87.1 ± 10.3kg) ingested either 1.87g of RC, 3.67g of CP (citrulline content 1.87g), 1.87g of RA, or 3.07g of AP (arginine content 1.87g) and performed 3 sets of 15 arm curls at 30 and 120 minutes post-supplementation. Brachial artery vessel diameter (VD) and blood flow volume (BFV) were measured via Doppler ultrasound at 0, 3, and 6 minutes post-exercise, corresponding to 30 (30P), 33 (33P), 36 (36P), 120 (120P), 123 (123P), and 126 (126P) minutes post-supplementation. Measurements were compared with both resting baseline (no treatment, no exercise) and active control (no treatment, exercise) values. Raw data were analyzed for all group, time, and group × time interactions using 2-way repeated-measures ANOVA. Delta values were analyzed using dependent T-tests. Alpha was predetermined at p < 0.05.

## Results

A significant (p < 0.05) group × time effect was present for VD, which significantly increased in CP versus RA from active baseline to 33P (CP: 0.57 ± 0.05; RA: 0.55 ± 0.05cm). Although, no effects for BFV were observed (p > 0.05). No differences were found between delta values for CP and AP nor between delta values for RC and RA or AP for VD (p > 0.05). However, VD delta values for CP were significantly (p < 0.05) greater than for RA at 33P (CP: +0.04 ± 0.03; RA: +0.02 ± 0.02cm) and 36P (CP: +0.04 ± 0.02; +0.02 ± 0.02cm) compared to active controls. A significantly (p < 0.05) greater change in BFV for the CP and RC treatments versus the RA treatment were observed at 33P (CP: +62.6 ± 155.8; RC: +57.6 ± 145.3; RA: -26.4 ± 137.4mL/min) compared to active control values. Conversely, significantly (p < 0.05) greater delta values for BFV were observed for AP over CP at 126P (AP: -5.6 ± 90.8; CP: -50.2 ± 74.7mL/min) compared to active controls.

## Conclusions

Collectively, citrulline-based ingredients appear to be more effective than arginine-based ingredients for modulating vasodilation and blood flow. The whey peptide bound state may positively influence the effects of supplementation.

